# Genome-wide association analysis of stalk biomass and anatomical traits in maize

**DOI:** 10.1186/s12870-019-1653-x

**Published:** 2019-01-31

**Authors:** Mona Mazaheri, Marlies Heckwolf, Brieanne Vaillancourt, Joseph L. Gage, Brett Burdo, Sven Heckwolf, Kerrie Barry, Anna Lipzen, Camila Bastos Ribeiro, Thomas J. Y. Kono, Heidi F. Kaeppler, Edgar P. Spalding, Candice N. Hirsch, C. Robin Buell, Natalia de Leon, Shawn M. Kaeppler

**Affiliations:** 1 Department of Agronomy, University of Wisconsin, Madison, WI 53706 USA; 2 Department of Energy, Great Lakes Bioenergy Research Center, University of Wisconsin, Madison, WI 53706 USA; 3 Department of Plant Biology, Michigan State University, East Lansing, MI 48824 USA; 4 Department of Energy, Great Lakes Bioenergy Research Center, Michigan State University, East Lansing, MI 48824 USA; 5 Department of Botany, University of Wisconsin, Madison, WI 53706 USA; 6 Department of Energy, Joint Genome Institute, Walnut Creek, California, 94598 USA; 7 Genótika Super Sementes. Colonizador Ênio Pipino - St. Industrial Sul, Sinop, MT 78550-098 Brazil; 8 Department of Agronomy and Plant Genetics, University of Minnesota, 1991 Upper Buford Circle, St Paul, MN 55108 USA; 9 Plant Resilience Institute, Michigan State University, East Lansing, MI 48824 USA; 10 Present address: Minnesota Supercomputing Institute, 117 Pleasant Street SE, Minneapolis, MN 55455 USA

**Keywords:** Maize, Stover, Stalk, Plant height, Rind, Vascular bundle, Genome-wide association, *Zmm22*

## Abstract

**Background:**

Maize stover is an important source of crop residues and a promising sustainable energy source in the United States. Stalk is the main component of stover, representing about half of stover dry weight. Characterization of genetic determinants of stalk traits provide a foundation to optimize maize stover as a biofuel feedstock. We investigated maize natural genetic variation in genome-wide association studies (GWAS) to detect candidate genes associated with traits related to stalk biomass (stalk diameter and plant height) and stalk anatomy (rind thickness, vascular bundle density and area).

**Results:**

Using a panel of 942 diverse inbred lines, 899,784 RNA-Seq derived single nucleotide polymorphism (SNP) markers were identified. Stalk traits were measured on 800 members of the panel in replicated field trials across years. GWAS revealed 16 candidate genes associated with four stalk traits. Most of the detected candidate genes were involved in fundamental cellular functions, such as regulation of gene expression and cell cycle progression. Two of the regulatory genes (*Zmm22* and an ortholog of *Fpa*) that were associated with plant height were previously shown to be involved in regulating the vegetative to floral transition. The association of *Zmm22* with plant height was confirmed using a transgenic approach. Transgenic lines with increased expression of *Zmm22* showed a significant decrease in plant height as well as tassel branch number, indicating a pleiotropic effect of *Zmm22*.

**Conclusion:**

Substantial heritable variation was observed in the association panel for stalk traits, indicating a large potential for improving useful stalk traits in breeding programs. Genome-wide association analyses detected several candidate genes associated with multiple traits, suggesting common regulatory elements underlie various stalk traits. Results of this study provide insights into the genetic control of maize stalk anatomy and biomass.

**Electronic supplementary material:**

The online version of this article (10.1186/s12870-019-1653-x) contains supplementary material, which is available to authorized users.

## Background

Economic and environmental crises caused by dependence on fossil fuels have prompted the use of alternative energy sources in recent decades. Agricultural crop residues represent an abundant and renewable lignocellulosic biomass resource and a promising sustainable feedstock to replace fossil fuels [1, 2]. Moreover, utilizing agricultural residues to produce biofuels would add extra value to growers of food and feed crops. Maize residues remaining after grain harvest, known as stover, is a predominant source of agricultural crop residues. At a 50% harvest index, 363 million tons of dry stover are estimated to have been produced in 2017, accounting for more than half of the total major crop residues in the United States (U.S.) [3]. Utilizing stover as a biofuel feedstock would establish maize as a multi-purpose crop for the production of not only food and feed, but also fuel.

Due to the large acreage of maize grown in the U.S., even a slight increase (5%) in stover yield per acre could potentially result in the production of an additional 621 million gallons of ethanol per year by increasing the total available lignocellulosic biomass [4]. It is predicted that stover yield would need to be increased by 30% by 2030 to meet the goal of replacing 30% of transportation fuels with biofuels [5]. Unlike other crops, biomass and grain yield are significantly correlated in maize [6]. This suggests that increasing stover biomass does not compromise grain yield for food. Improving stover yield requires characterizing the genetic structure underlying stover-related traits. Stalk is the main component of stover, representing approximately 46% of the stover dry weight [7]. Thus, stalk length (plant height) and stalk diameter are key traits determining overall stover yield.

Plant height is highly correlated with biomass yield [8]. In the past, maize breeders selected for reduced plant height because shorter plants are more resistant to lodging [9]. In contrast, optimizing stover for biofuel production may shift this paradigm to breed for taller plants [4, 10]. Quantitative trait loci (QTL) mapping and genome-wide association studies (GWAS) have detected numerous plant height loci in maize [11–16], among which, several genes have been validated and characterized including *Dwarf3* [17], *Brachytic2* [18], *Nana plant1* [19], *Dwarf 8*, *Dwarf 9* [20], and *Ga3ox2* [21]. Unlike plant height, little is known about genetic loci underlying maize stalk diameter. A QTL mapping study using 294 markers detected ten small effect stalk diameter loci. The large intervals of detected loci prevented accurate co-localization of detected QTLs and the underlying genes [22]. Another QTL study detected a major stalk diameter QTL within a 2.5 Mb interval on chromosome 5, explaining 21% of the variation [23].

Improving stalk biomass also necessitates characterization of stalk anatomy. The high lignin content of secondary cell walls makes stover recalcitrant to enzymatic digestion of polysaccharides into fermentable sugars [24, 25]. Maize stalk is comprised of rind and pith sections, each composed of tissues with different levels of cell wall lignification. For example, rind and sclerenchyma cells surrounding pith vascular bundles are more lignified compared to other tissues found in stalk, such as pith in the center of stalk [26–28]. Considering these factors, stalk anatomy is directly related to the lignin content, and therefor correlated to stalk digestibility. A study of 22 maize inbreds, including brown midrib mutants (*bm3*) and their corresponding wild types, showed that 89% of phenotypic variation for stalk digestibility can be explained by a combination of stalk anatomy and composition [29]. Therefore, characterizing stalk anatomical traits and their underlying genetic networks would be an important step to improve stalk digestibility. Previously, laborious microscopic measurements have hindered exploration of diversity for stalk anatomical traits. However, recent developments in high-throughput image analysis tools have enabled large-scale analysis of stalk anatomical traits including rind thickness, vascular bundle density, and vascular bundle area [30].

GWAS is a powerful approach to associate natural phenotypic diversity with underlying genome sequence variants. In maize, the abundance of single nucleotide polymorphism (SNP) data and rapid linkage disequilibrium (LD) decay (~ 2 kb) enable the detection of associated genomic regions at the single-gene resolution level [31]. GWAS has been successfully applied to dissect the genetic composition of several complex traits in maize including flowering time [32], leaf architecture [33], disease resistance [34, 35], and kernel composition [36]. Previously, the Wisconsin Diversity (WiDiv) panel was characterized for association studies in maize [37, 38]. This panel included 503 inbreds adapted to the upper Midwest region of the U.S. and was genotyped with 451,066 SNPs generated from seedling RNA-Seq reads [38]. The main objectives of the present study were to expand the population size and genotypic data of the WiDiv panel and utilize the expanded panel to detect candidate genes associated with stalk biomass (plant height and stalk diameter) and stalk anatomy (rind thickness, vascular bundle density, and vascular bundle area).

## Results

### Expanded WiDiv panel

The previous WiDiv population [37, 38] was expanded to a panel of 942 inbred lines (WiDiv-942). The WiDiv-942 panel includes a diverse set of public, expired plant variety protection (exPVP), and germplasm enhancement of maize (GEM)-derived inbreds. This panel represents some of the major North American field corn heterotic groups, including stiff stalk (SS), non-stiff stalk (NSS), and Iodent (IDT), as well as sweet corn, popcorn, and tropical inbreds. This expanded panel also has unselected inbreds from synthetic populations and landraces including 54 inbreds derived from Iowa stiff stalk cycle 0 (BSSSC0) synthetic population. Pedigree information for the WiDiv-942 panel is provided in Additional file 1.

**Fig. 1:**
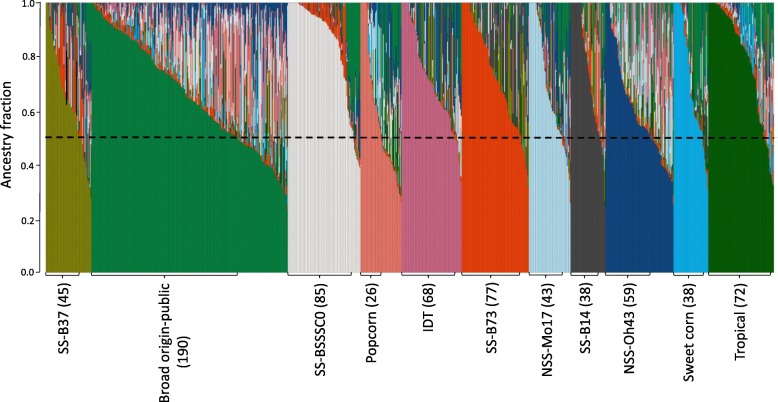
Graphical representation for the WiDiv-942 subpopulations. Population structure was determined using ADMIXTURE model at subpopulation number = 11. Number of inbreds within each subpopulation is shown in parenthesis. A total of 201 inbreds with less than 0.5 ancestry fraction were classified as “mixed”

A total of 899,784 SNPs was identified using RNA-Seq data from whole seedlings of each member of the WiDiv-942 panel. The software program Admixture 1.23 [39] classified the WiDiv-942 panel into subpopulations using a subset of 93,991 SNPs that were pruned based on a pairwise LD threshold of *r*^*2*^ = 0.1. Each subpopulation was labeled based on the pedigree of the majority of the inbreds within that subpopulation. According to this classification, the WiDiv-942 panel was divided into four SS subpopulations, two NSS subpopulations, one subpopulation of broad origin-public lines, one IDT, one sweet corn, one popcorn, and one tropical subpopulation. The four SS subpopulations were B37, B73, B14, and BSSSC0 types and the two NSS subpopulations were Mo17 and Oh43 types. A total of 201 inbreds with less than 0.5 probability of membership to any of the subpopulations was classified as “mixed” (Fig. 1, Additional file 1).

### Evaluating stalk traits

**Fig. 2:**
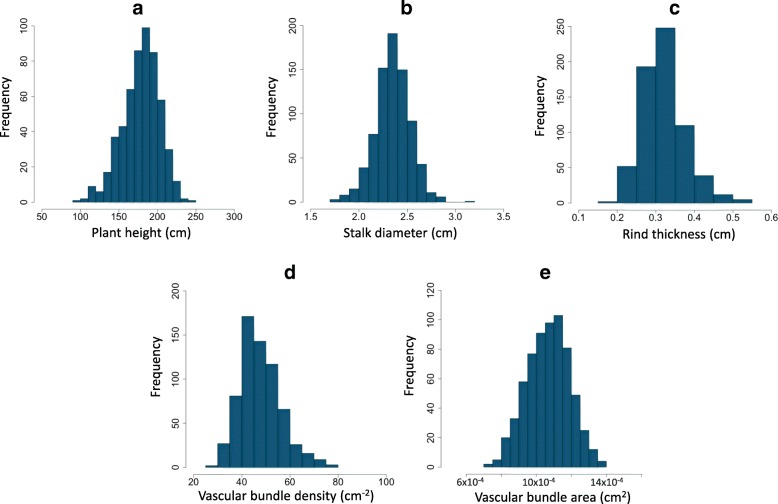
Distribution of **a**) plant height, **b**) stalk diameter, **c**) rind thickness, **d**) vascular bundle density, and **e**) vascular bundle area traits in the WiDiv-942 panel

**Fig. 3:**
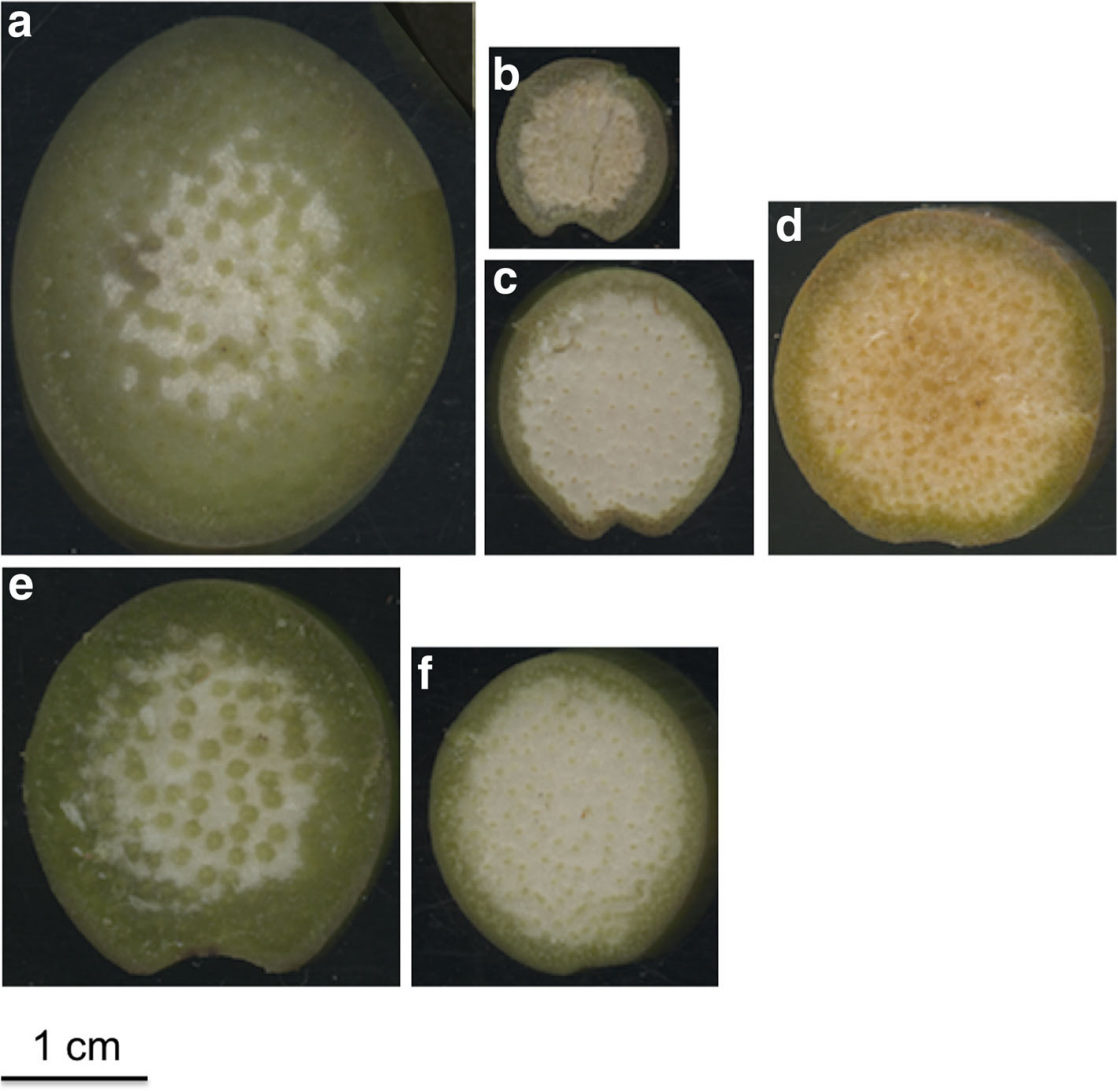
Scan images of the third above ground internodes of a sample of inbreds with extreme stalk trait measurements **a**) Inbred B104: large stalk diameter, thick rind, and low vascular bundle density, **b**) Inbred W85: narrow stalk, **c**) Inbred W611S: thin rind, **d**) Inbred 52220: high vascular bundle density, **e**) Inbred CO117: large vascular bundle area, and **f**) Inbred CSJ3: small vascular bundle area

Stalk traits were measured in 800 members of the WiDiv-942 panel. Stalk diameter and plant height were measured in five and three years, respectively. Rind thickness, vascular bundle density, and vascular bundle area were measured in two years. The measurement of each trait for a given inbred was estimated by adding the best linear unbiased prediction (BLUP) value to the mean of that trait across the population (Additional file 1). Substantial variation was observed for stalk traits (Figs. 2 and 3, Table 1). Vascular bundle density showed the highest variation, ranging from 25.59 cm^− 2^ to 77.45 cm^− 2^ (3.03-fold difference). Rind thickness had the next greatest variation, ranging from 0.19 cm to 0.54 cm (2.84-fold difference). Plant height, vascular bundle area, and stalk diameter measurements ranged from 99.31 cm to 242.86 cm (2.45-fold difference), from 0.73 × 10^− 3^ cm^2^ to 1.38 × 10^− 3^ cm^2^ (1.89-fold variation), and from 1.78 cm to 3.11 cm (1.75-fold difference), respectively (Table 1). A relatively high heritability was observed for all traits. The most heritable trait was plant height (${\widehat{h}}^2$=0.93), followed by stalk diameter (${\widehat{h}}^2$=0.87), vascular bundle area (${\widehat{h}}^2$=0.85), and vascular bundle density (${\widehat{h}}^2$=0.73). Rind thickness, with ${\widehat{h}}^2=0.58,$ was the least heritable of the traits included in this study. Each of the traits showed significant correlations across environments. For each trait, the data were significantly correlated across the years. Analysis of variance detected a significant genotype-by-environment interaction for plant height and stalk diameter, whereas the genotype-by-environment interaction for rind thickness, vascular bundle area, and vascular bundle density was not significant.

**Table 1:** Mean and variation of stalk traits in the WiDiv-942 panel

	Mean ± SD^a^	Range
Plant height (cm)	179.14 ± 29.29	99.31–242.86
Stalk diameter (cm)	2.35 ± 0.36	1.78–3.11
Rind thickness (cm)	0.32 ± 0.15	0.19–0.54
Vascular bundle density (cm^− 2^)	47.84 ± 12.52	25.59–77.45
Vascular bundle area (cm^2^)	10.72 × 10^− 4^ ± 2.32 × 10^− 4^	7.34 × 10^− 4^-13.86 × 10^− 4^

^a^Standard deviation

**Fig. 4:**
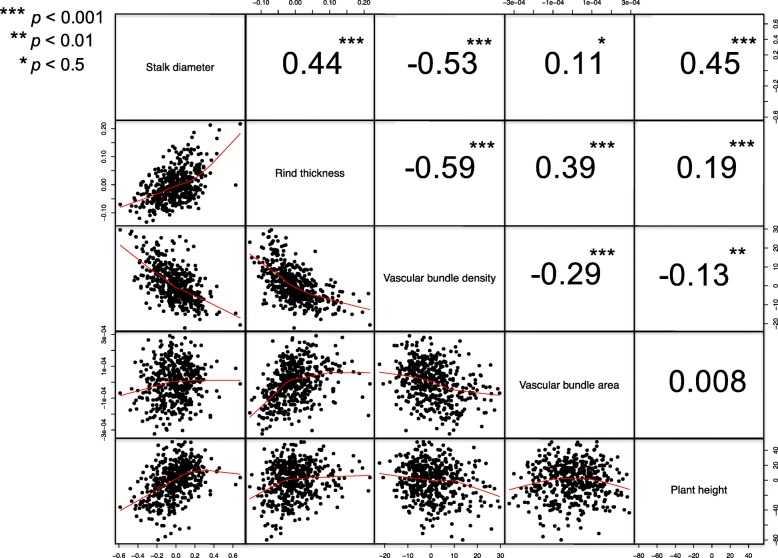
Correlation between stalk traits. Spearman correlation was calculated between BLUP values. Red lines represent non-linear correlations between the traits

Significant correlations were detected among the measured stalk traits, except for the correlation between plant height and vascular bundle area (*ρ* = 0.008) (Fig. 4). Vascular bundle density had significant negative correlations with stalk diameter, rind thickness, vascular bundle area, and plant height, whereas the correlations among the remaining traits were positive. Strong correlations were observed among three traits: stalk diameter, rind thickness, and vascular bundle density, in which stalk diameter and rind thickness were positively correlated and vascular bundle density was negatively correlated with stalk diameter and rind thickness. This suggests that inbreds with wide stalks often have thick rinds and low vascular bundle density. This trend was exemplified by the inbred B104 that had stalk diameter, rind thickness, and vascular bundle density measurements all within the extreme 10% of measurements (Fig. 3a).

### Genome-wide association analysis

**Fig. 5:**
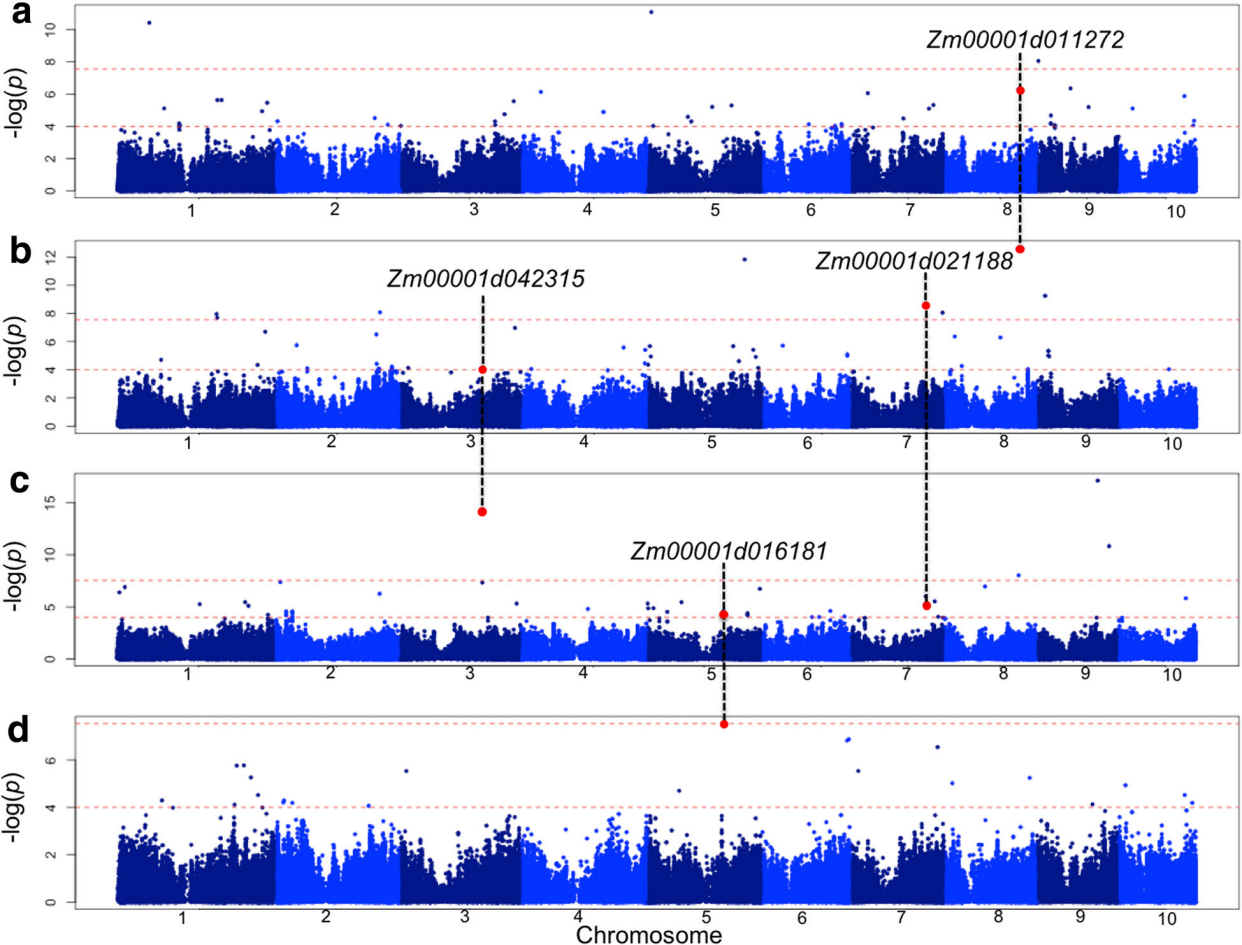
Manhattan plots of GWAS results for **a**) vascular bundle density, **b**) stalk diameter, **c**) plant height, and **d**) rind thickness. Red dashed lines correspond to the Bonferroni threshold (−log(*p*) = 7.55) and the threshold of –log(*p*) = 4. Red dots indicate significant SNPs (−log(*p*) > 7.55) associated with one trait that also showed association signals (−log(*p*) > 4) with other stalk traits. Candidate genes corresponding to these SNPs are shown above vertical black dashed lines

Imputed data of the 899,784 detected SNPs was used for GWAS of the stalk traits. The proportion of missing data after imputation was 0.002. Imputation accuracy was evaluated for 56 inbred lines by comparing SNP calls at imputed sites to high quality whole genome resequencing SNP calls. The imputed and whole genome resequencing SNP datasets had 463,187 sites in common across all ten chromosomes; the average imputation accuracy was 0.92 over the 56 inbred lines. While this value may seem somewhat low, it compares data from different analysis pipelines and different samples. It is reported here as we believe it is helpful for the community to understand inherent limitations of this, and similar, datasets when comparing across studies. GWAS for stalk biomass and anatomical traits were performed using the program FarmCPU [40]. The kinship (K) model was used in the association analyses to correct for potential false positive associations related to familial relatedness. The model fitness was confirmed by inspecting quantile-quantile (Q-Q) plots that compared the observed and expected *p*-values under the null hypothesis of no associations (Additional file 2). A total of 4, 8, 1, and 3 loci were associated with plant height, stalk diameter, rind thickness, and vascular bundle density, respectively, based on a genome-wide corrected Bonferroni threshold of –log(*p*) = 7.55 (Fig. 5, Table 2). We did not detect any significant associations for vascular bundle area at the –log(*p*) = 7.55 threshold (Additional file 3). This could be an indication that vascular bundle area is genetically more complex and is controlled by small effect genes compared to other stalk traits measured in this study.

**Table 2:** SNPs and corresponding candidate genes associated with plant height (PH), stalk diameter (SD), rind thickness (RT), and vascular bundle density (BD)

Trait	SNP ID	MAF	-log(*p*)	Effect size (%)	Gene containing the SNP	Candidate gene	SNP position relative to the candidate gene^2^	Candidate gene annotation
PH	rs9_119248765^1^	0.14	17.15*^3^	4.31	*Zm00001d047117*	*Zm00001d047121*	−24.05 kb	NAD-dependent protein deacetylase Srt2 (Regulatory gene)
PH SD	rs3_160558889	0.23 0.24	14.34* 4.10^4^	3.19 2.13	*Zm00001d042314*	*Zm00001d042315 (Zmm22)*	−32.60 kb	MADS box transcription factor 69 (Regulatory gene)
PH	rs9_141541062	0.37	10.83*	2.23	*Zm00001d047779*	*Zm00001d047780*	−26.53 kb	ABC transporter (Auxin transporter)
PH	rs8_145826594	0.49	8.06*	1.67	*Zm00001d011285 (Lhcb1)*	*Zm00001d011285 (Lhcb1)*	0	Light harvesting chlorophyll protein 1 (Photosynthesis related)
SD BD	rs8_1453700352	0.25 0.23	12.67*^3^ 6.32	3.83 2.60	*Zm00001d011272*	*Zm00001d011272*	0	CASC3/Barentsz eIF4AIII binding (Regulatory gene)
SD	rs5_189094902	0.20	11.83*	4.25	*Zm00001d017213*	*Zm00001d017213*	0	DUF 4378
SD	rs9_15314439	0.19	9.25*	3.40	*Zm00001d045179*	*Zm00001d045180*	−8.26 kb	Cdk-activating kinase assembly factor MAT1 (Cell division regulator)
SD PH	rs7_145418275 rs7_145449766	0.29 0.50	8.64* 5.24	2.98 1.29	*Zm00001d021188 (Fpa* ortholog*) Zm00001d021192*	*Zm00001d021188 (Fpa* ortholog*)*	0 + 31.23 kb	Flowering time control protein (Regulatory gene)
SD	rs2_204222535	0.11	8.07*	4.25	*Zm00001d006308*	*Zm00001d006308*	0	Protein kinase (Regulatory gene)
SD	rs7_178139632	0.05	8.05*	5.53	*Zm00001d022460*	*Zm00001d022460*	0	Pectinesterase (Cell wall biosynthesis)
SD	rs1_191586515	0.40	7.95*	2.55	*Zm00001d031485*	*Zm00001d031485*	0	Cdc2 cell division control protein (Cell division regulator)
SD	rs1_192993003	0.10	7.69*	3.83	*Zm00001d031531*	*Zm00001d031531*	0	DUF1666
RT PH	rs5_148922178 rs5_148941136	0.06 0.19	7.55* 4.58	6.25 1.62	*Zm00001d016181 Zm00001d016182*	*Zm00001d016181*	0 + 16.22 kb	Glucuronosyltransferase (Cell wall biosynthesis)
BD	rs5_7499864	0.20	11.10*	4.05	*Zm00001d013267 (Tlk2)*	*Zm00001d013267 (Tlk2)*	0	TOUSLED- like kinase 2 (Cell division regulator)
BD	rs1_61042634	0.08	10.43*	5.69	*Zm00001d029177 (Nsn1* ortholog)	*Zm00001d029177 (Nsn1* ortholog)	0	Nucleolar GTP-binding protein 2 (Regulatory gene)
BD	rs9_2777619	0.22	8.05*	3.30	*Zm00001d044802 (Rop5)*	*Zm00001d044802 (Rop5)*	0	Rop family GTPase (Regulatory gene)

^1^Values after “rs” and “_” represent SNP chromosome number and physical position (bp), respectively

^2^SNPs upstream and downstream of candidate genes are specified with “–” and “+”, respectively. “0” indicates that SNPs are located within corresponding candidate gens

^3^Significance according to the Bonferroni genomic threshold (−log(*p*) = 7.55)

^4^Values not specified with “*” were above the –log(*p*) = 4 threshold but did not reach the Bonferroni genomic threshold

### Candidate genes associated with stalk traits

Genes/gene models containing or adjacent to the SNPs above the Bonferroni significance threshold were selected as candidate genes (Table 2). When a gene harboring a significant SNP had functional annotation or expression information inconsistent with stalk development, for example encoding a cortical protein specifically in roots, the adjacent gene with the most relevant annotation was selected as the candidate gene. LD between significant SNPs and adjacent candidate genes were validated using Haploview software [41] (Additional file 4).

Two candidate genes were annotated as domain with unknown function (DUF) whereas the remaining candidate genes had putative functions consistent with regulating gene expression, cell division, cell wall biosynthesis, photosynthesis, and auxin transport. These cellular functions support potential association of detected candidate genes with the traits described in this study. Based on the maize gene atlas [42, 43], most candidate genes were expressed in all tissues throughout maize development, except for a light harvesting chlorophyll gene (*Zm00001d011285*) that was specifically expressed in leaves (Additional file 5).

To determine whether detected candidate genes were associated with multiple stalk traits, we searched for SNPs above a threshold of –log(*p*) = 4.00 that were located within or adjacent to the candidate genes. The –log(*p*) = 4 threshold was selected because it appeared to separate the top SNPs from the mass of non-associated SNPs in the Manhattan plots (Fig. 5). Using this threshold resulted in identifying associations that did not reach the Bonferroni significance threshold due to small effect size (Table 2). We detected four candidate genes associated with one stalk trait, at the Bonferroni level, that also showed association signals at the –log(*p*) > 4 level with other stalk traits. (Fig. 5, Table 2). Two flowering time candidate genes (*Zmm22* and an *Fpa* ortholog) were associated with both stalk diameter and plant height, a putative regulatory gene (*Zm00001d011272)* was associated with stalk diameter and vascular bundle density, and a putative cell wall biosynthesis gene model (*Zm00001d016181*) was associated with plant height and rind thickness.

### Ectopic expression of *Zmm22*

*Zmm22* was one of the most significant plant height candidate genes that also showed association with stalk diameter, suggesting that *Zmm22* could be a promising candidate gene for improving stover biomass. The association of *Zmm22* with plant height was supported by genetically modified plants that over-expressed the endogenous gene. Three events of *Zmm22* over-expression were developed. Transgenic plants were distinguished from their non-transgenic siblings (control) by expression of the red fluorescent protein (RFP) and resistance to the herbicide bialaphos. In addition, semi-quantitative PCR (sqPCR) analysis confirmed *Zmm22* over-expression in the transgenic lines (Additional file 6). Agronomic traits were measured in the T_2_ generation of transgenic and control plants in replicated field trials.

Over-expression of *Zmm22* not only reduced plant height, but it also reduced stalk diameter and tassel branch number (Table 3). One particular transgenic event (Event 1) had the most significant reduction of overall plant size. Transgenic lines within this event showed an average 19, 11, 35, 50, 37, and 10% reduction in plant height, stalk diameter, tassel branch number, stover yield, cob length, and cob width, respectively, compared to wild-type siblings. Plant height, stalk diameter, tassel branch number, and stover yield were also significantly reduced in transgenic lines of Event 2. Transgenic lines within Event 3 showed a significant reduction in plant height and tassel branch number. Evaluation of the T_1_ generation of Event 1 and Event 2 plants also showed a significant reduction in plant height and stalk diameter (Additional file 7), indicating the stability of the *Zmm22* over-expression effect.

**Table 3:** Comparing the average of agronomic traits for three *Zmm22* over-expression events to that for their non-transgenic siblings (control). A * indicates that the control is significantly different from the transgenic

	Event 1		Event 2		Event 3	
	Control	Transgenic	Control	Transgenic	Control	Transgenic
Number of individuals	12	10	10	9	10	12
Plant height (cm)	204.50	165.56*^1^	209.50	193.89*	210.56	178.64*
Stalk diameter (cm)	2.42	2.15*	2.42	2.19*	2.06	2.04 ns
Tassel branch number	11.10	7.22*	12.30	8.56*	10.25	7.64*
Stover yield (gr)	116.13	60.79*	116.50	84.93*	85.64	75.94 ns
Cob length (cm)	13.46	9.65*	12.51	11.45 ns	11.91	11.44 ns
Cob width (cm)	2.53	2.29*	2.47	2.59 ns	2.40	2.45 ns
Ear height (cm)	108.00	61.88*	117.00	86.25*	124.00	74.38*
Below ear internode number	8.30	5.44*	8.60	6.50*	8.60	5.57*
Below ear internode length (cm)	13.05	11.47*	13.61	13.29 ns	14.45	13.45*
Above ear height (cm)	96.50	125.00*	92.50	117.22*	86.67	103.57*
Above ear node number	6.70	6.71 ns^2^	6.30	6.75 ns	6.67	6.71 ns
Above ear internode length (cm)	14.45	16.29*	14.70	15.98 ns	12.99	15.46*

^1^Significance at *p* < 0.05

^2^Non-significant

We measured node number and internode length below and above the primary ear, ear height, and above ear height to determine the effect of *Zmm22* on overall plant height components. Ear height and below ear node number showed a significant decrease in the three transgenic events. In contrast, above ear height increased significantly in the three events. Transgenic lines within Event 1 and Event 3 had a significant decrease in below ear internode length and a significant increase in above ear internode length. We did not detect any significant difference in kernel number and kernel weight between transgenic and non-transgenic lines at *p* < 0.05.

## Discussion

We exploited the genetic variation of the WiDiv-942 panel in GWAS and detected 16 candidate genes associated with stalk biomass and anatomical traits. Annotation information and maize atlas gene expression data suggested that most of the detected candidate genes associated with stalk traits may control multiple traits during maize growth and development. This is supported by a putative cell wall biosynthesis gene (*Zm0001d016181*) and three putative regulatory genes (*Zmm22*, an *Fpa* ortholog, and *Zm0001d011272*) being associated with two stalk traits (Fig. 5). The effect of regulatory genes on multiple traits has been long established [44] and indicates that they coordinate the expression of multiple genes. The following sections describe candidate genes associated with each stalk traits.

### Plant height

Four candidate genes associated with plant height were detected. The most significant SNP associated with plant height was located 24.05 kb upstream of a regulatory gene (*Srt2*), which is involved in chromatin silencing [45, 46]. Down-regulation of *Srt2* in rice was related to an increased level of histone acetylation and resulted in multiple phenotypes including accelerated leaf senescence, shortening of internodes, dwarfism, and small grains [47]. *Srt2* did not contain any single polymorphism in our SNP dataset. The lack of allelic variation could indicate that *Srt2* is associated with plant height through variation in expression level and that causative SNP might be located in the promoter region.

The *Zmm22* region was the second most significant locus associated with plant height and was also associated with stalk diameter. This region was previously associated with plant height in a separate association panel [16]. *Zmm22,* also known as *ZmMADS69,* is a flowering time gene in maize [38, 48] and encodes a member of the MADS-box transcription factor family, a common key player in the evolution of the plant reproductive system [49]. The rice *Zmm22* ortholog, *OsMADS51*, activates a signaling cascade that leads to the expression of the florigen signal, and consequently induces flowering [50]. Several studies have reported a conserved floral transition regulatory network between rice and maize [51–53], suggesting that *Zmm22* might induce flowering in maize by triggering florigen expression. Using transgenics, we showed that *Zmm22* controls plant height, stalk diameter, tassel branch number, and cob size. This result was in agreement with a recent study that confirmed the pleiotropic effect of this flowering time gene on plant height, stalk diameter, and tassel and ear size [48]. Another study identified the *Zmm22* region as a target of selection during maize domestication [22]. This region was identified as the location of many QTLs for numerous traits including barren nodes, internode length, plant height, branch length, hardiness of glume, flowering time, ear diameter, tassel length, and tassel branch number [22].

The third plant height candidate gene was *Zm00001d047780*. This gene encodes an ATP-binding cassette (ABC) transporter similar to mammalian multi drug resistance class of P-Glycoprotein (MDR/PGP) transporters. PGPs in plants facilitate polar movement of auxin and are essential for auxin-mediated developmental processes [54, 55]. Interruption of expression of *PGP* genes have pleiotropic effects on several traits including plant height [18, 56]. A *PGP* mutant (*Dw3*) has been used extensively in sorghum breeding programs to reduce plant height [18]. Loss of function of the same gene in maize *Brachytic2* (*Br2*) mutants interrupted polar auxin transport in the stalk and resulted in shortening of lower stalk internodes, increase in stalk diameter, and alteration of vascular bundle pattern [18].

*Lhcb1,* another plant height candidate gene, encodes a light harvesting chlorophyll binding (Lhcb) protein. The Lhcb1 family is a major part of the light harvesting antenna complex that transfers the photosynthetic electrons from sunlight to photosynthetic complexes [57]. Mutants containing *Lhcb1* loss of function alleles showed a reduced fitness when evaluated under field conditions [58]. Overexpression of *Lhcb1* in tobacco increased cell volume, biomass, and seed weight, and delayed flowering time under low irradiance level [59].

### Stalk diameter

Eight candidate genes associated with stalk diameter were detected. Three of the candidate gene models (*Zm000*01d011*272, Zm00001d021188,* and *Zm00001d006308*) encoded transcriptional and post-transcriptional regulatory elements. *Zm00001d011272* contained the most significant SNP associated with stalk diameter and was also associated with vascular bundle density. The product of this gene is similar to the binding domain of a post-transcriptional regulatory family in metazoa (CASC3/Barentsz eIF4AIII) [60]. An ortholog of *Zm00001d011272* in Arabidopsis (*AT1G80000*) was reported as a candidate gene underlying cell proliferation and organogenesis through epigenetic silencing of homeotic genes [61]. *Zm00001d021188* was another stalk diameter candidate gene with a regulatory function. This gene was also adjacent to a SNP associated with plant height. An ortholog of this gene in Brachypodium *(Fpa*) is a well-characterized flowering time gene. *Fpa* silences a wide range of genes including a flowering inhibitor gene (*Flc*) [62, 63]. A mutation within an ortholog of this gene in Arabidopsis not only delayed flowering time but also resulted in a small rosette diameter, indicating a wide range of function for this gene [62]. *Zm00001d006308,* another putative regulatory gene, encodes a kinase protein that is orthologous to an alternative splicing modulator in Arabidopsis (*Srpk*) [64]. Alternative splicing regulators impact various cellular functions through modifying the splicing pattern of pre-mRNAs and altering the translation products [65]. Over-expression of alternative splicing regulators has been linked to multiple plant morphology and physiology processes in Arabidopsis [66, 67].

Two of the stalk diameter candidate gene models (*Zm00001d045180* and *Zm00001d031485*) encode cell cycle regulatory proteins. Cell cycle progression is the fundamental basis for cell proliferation, growth, and development and is regulated by cyclin-dependent kinase (Cdk) complex [68]. The *Zm00001d045180* gene product (MAT1) is involved in activating Cdk, and *Zm00001d031485* encodes a cell division control protein (Cdc2) within the Cdk complex [69, 70].

*Zm00001d022460*, another stalk diameter candidate gene, encodes a putative pectinesterase (PE) enzyme, a cell wall associated enzyme involved in demethylation of galacturonyl residue of pectin. PE impacts cell wall expansion and other cell wall physical properties [71] and has been linked to various biological functions including stem elongation [72].

### Rind thickness

*Zm00001d016181* was the only candidate gene with a significant association with rind thickness. This gene was also adjacent to a SNP associated with plant height. *Zm00001d016181* encodes an enzyme with glucuronyltransferase activity. Glucuronyltransferase enzymes are essential for biosynthesis of pectin and xylon components of plant cell walls [73, 74]. Mutations in the glucuronyltransferase related genes (*IRX10* and *IRX10-L*) were linked to reduced stem cell wall thickness, narrow stem, and dwarfism in *Arabidopsis* [74].

### Vascular bundle density

Three vascular bundle density candidate genes were identified. One of the candidate genes (*Zm00001d013267*) encoded a member of the serine/threonine Tousled-like kinases (TLK) family that regulates chromatin condensation during mitosis [75]. A *TLK* loss-of-function mutation had a pleiotropic effect on the morphology of leaf and flower in Arabidopsis by potentially inhibiting cell division and tissue expansion [76, 77]. Another vascular bundle density candidate gene (*Zm00001d029177)* encoded a nucleolar GTPase enzyme. A homolog of this gene in Arabidopsis (*Nsn1*) regulates the expression of a flowering gene (*AG*). *Nsn1* loss of function was related to disruption in flower formation and vegetative growth [78]. Finally, *Zm00001d044802* encodes a member of the Rho-related GTPase (Rop) family. Members of the Rop family regulate a wide range of biological processes including vegetative growth, cell morphogenesis, and signal transduction [79]. Rop proteins have been related to xylem cell wall biosynthesis and vascular bundle development [80, 81].

### Association of flowering time genes with stalk traits

Previous studies have reported pleiotropic effect of flowering time genes, *Zcn8*, *Zfl1*, and *Zfl2*, on a variety of maize morphological traits [82, 83]. Here, results from our association studies detected three flowering time candidate genes (*Nsn1* and *Fpa* orthologs and *Zmm22*) associated with stalk traits. The *Nsn1* ortholog was associated with vascular bundle density whereas the *Fpa* ortholog and *Zmm22* were associated with both stalk diameter and plant height. Genetic correlation between flowering time and plant height in maize has been indicated in previous studies [16, 84] and is consistent with vegetative apical growth being terminated during the emergence of male inflorescence, or tassel [85]. However, the association of flowering time genes with vascular bundle density and stalk diameter was unexpected, suggesting a pleiotropic effect of the three flowering time genes (*Fpa* and *Nsn1* orthologs and *Zmm22*) on stalk traits. This was consistent with a study that showed a flowering time gene (*Ghd7*) has a large effect on stem thickness and vascular bundles in rice [86]. *Fpa* and *Nsn1* orthologs and *Zmm22* have putative regulatory functions and are expressed in various tissues throughout the maize life cycle (Additional file 5), which support their association with multiple traits. Transformation studies confirmed the pleiotropic effect of *Zmm22,* showing that over-expression of this gene significantly reduces overall plant size.

The genetic correlation between flowering time and stalk traits was consistent with phenotypic correlation among traits. We observed an “early-flowering syndrome” in the WiDiv-942 population in which inbred lines that flowered early had a narrow stalk, thin rind, and dense vascular bundles (Additional file 8). These results suggest that improving stalk biomass will often result in delayed flowering, affecting adaptation and cultivar deployment. Further studies on genetic control of the early-flowering syndrome is required to decouple stalk traits and flowering time and to develop inbreds that have improved stalk biomass as well as flowering time consistent with their target deployment zone.

## Conclusions

We expanded an existing diversity panel and assembled a population of 942 inbred lines with diverse backgrounds. A total of 899,784 SNPs was developed from seedling RNA-Seq data. The generated SNP dataset is representative of genic sequence variation across a large number of diverse inbred lines. The expanded diversity panel and the SNP dataset developed in this study are valuable resources for future GWAS in maize. A large variation for stalk traits was observed in the population, ranging from 1.75-fold difference for stalk diameter to 3.03-fold difference for vascular bundle density. The large variation of stalk traits indicates a potential to utilize endogenous genetic diversity of inbred lines to improve stalk traits and overall biomass production in maize. We detected 16 candidate genes associated with stalk biomass and anatomical traits, several of which may control multiple traits. Two of the candidate genes (*Zmm22* and an *Fpa* ortholog) were flowering time genes that were associated with stalk diameter. Another flowering time candidate gene (an *Nsn1* ortholog) was found in association with vascular bundle density. A putative cell wall biosynthesis gene model (*Zm0001d016181*) was associated with plant height and rind thickness, and a putative regulatory gene (*Zm0001d01618)* was associated with stalk diameter and vascular bundle density. Manipulating candidate genes detected in this study may result in improving overall stover biomass and relevant stalk anatomical traits that have the potential to affect biomass compositional characteristics.

## Methods

### Expanding the WiDiv population

A set of 453 inbred lines were added to the previously developed WiDiv population [38]. Pedigree information of the expanded population was obtained by compiling information from multiple sources [37, 87, 88]. Total RNA of the 453 inbreds was extracted from whole seedlings at the V1 developmental stage as described previously [38]. RNA-Seq libraries were prepared using the Tru-Seq RNA-Seq kit (both stranded and non-stranded) and sequenced on the Illumina HiSeq platform generating 150–151 nt paired-end reads. Information of RNA-Seq reads, such as number of reads and SRA number, are provided in Additional file 1. Reads were treated as single end through the entire quality and mapping pipeline. Reads were mapped to spike-in sequences [89] and the UniVec database [90] using Bowtie version 0.12.7 [91] with default parameters. Libraries with more than 5% spike-in/UniVec sequences were removed. Reads were trimmed using Cutadapt v1.8 [92] to remove adaptors and low quality sequences using a quality score cutoff of 20; reads less than 30 nt were discarded. Libraries in which more than 20% of reads were removed were discarded. For read length consistency between the previous WiDiv panel and the added inbreds, sequence reads were trimmed to 100 nt using the FASTX_trimmer from the FASTX Toolkit v0.0.14 [93]. Poly A/T tails were removed using Cutadapt v1.8 [92] setting described above. Poly A/T tails with ≥20 nt length were removed up to four times at the 5′ or 3′ end, and the minimum read length was set to 30 nt. Libraries with less than five million reads after cleaning were discarded.

### Read alignment and assembly of novel transcripts

Cleaned reads were aligned to the version 4 maize B73 (AGPv4) reference sequence assembly (excluding mitochondrial and plastid sequence) [94] using Tophat2 v2.0.14 [95] and Bowtie2 v2.2.3 [91]. The minimum and maximum intron size was set to 5 bp and 60,000 bp, respectively, and novel indel detection was disabled. Other parameters were set to default. Unmapped bam files from Tophat output were converted to fastq using BEDTools v 2.25.0 [96] and separated into unmapped pairs and singletons. To identify novel transcripts within the inbreds and improve our variant calling, an assembly was made with these reads. First, the reads were normalized using the in silico read normalization utility provided by Trinity v2.2.0 [97]. Two rounds of normalization were run, one with paired end and the other with the singleton fastq files, with a maximum coverage of 30. Paired end data were run with the parameter --pairs_together. Since some datasets were not stranded, the normalized singletons were concatenated to the end of normalized data from read one. Trinity v2.2.0 [97] was run with a minimum k-mer count of 2 nt, minimum contig length of 500 bp, and a group pairs distance of 500 bp. The longest isoform per gene was selected. Assembled reads were aligned to AGPv4 including the mitochondrial and plastid sequence using GMAP v.2012-04-21 [98] with default parameters. Transcripts with more than 85% coverage and identity were discarded. Transcripts were filtered against megablastn Spike-in [89], megablastn Univec [90], blastn-short Illumina adapters (Oligonucleotide sequences, Illumina, Inc. 2016), megablastn NCBI-nt, and blastx NCBI-nr [99] using Blast+ v2.50 [100] with an e-value cutoff of 1e-5. Transcripts with > = 50% coverage of query or subject and > =95% identity to Spike-in or Univec were discarded. For the NCBI nt search, transcripts with > = 95% identity, > = 50% coverage of the subject or query, and a hit to a non-Viridiplantae were removed. Transcripts with > = 50% identity, > = 50% coverage of the query or subject, and non-Viridiplantae were removed based on the NCBI nr search. In addition, transcripts with a hit to Illumina adapters were removed. To reduce redundancy of Trinity transcripts, CD-HIT v4.6 [101] was run with a sequence identity threshold of 95%. Additional quality control of the Trinity assembly was performed by running Blastp against genomic sequences of AGPv4 [94], *Oryza sativa* v7 [102], *Sorghum bicolor* v3.1 [103], *Arabidopsis thaliana* (TAIR 10) [104], and *Brachypodium distachyon* v3.1 [105] using Blast+ v2.5.0 [100]. The TransDecoder.LongOrfs tool from Transdecoder [106] v3.0.1 was used to output the longest open reading frame for each Trinity transcript to use as the query in the above blastp.

### Detecting SNPs and imputing missing data

All cleaned reads were re-aligned to the B73 v4 reference genome and our set of novel Trinity assembled transcripts using Tophat2 v2.0.14 [95] and Bowtie2 v2.2.3 [91]. The minimum and maximum intron size were set to 5 bp and 60,000 bp, respectively, and other parameters were set to default. On average, 87.5% of the reads aligned to the AGPv4 reference genome plus our Trinity assembled transcripts, of which, 89.1% were uniquely mapping and 10.9% were multi-mapping (Additional file 1). SNPs were called from the alignments using SAMTools v0.1.18 [107] by creating a mpileup file with BAQ computation disabled, indel calling disabled, and a MAPQ filter of 50; calls with a base quality of > 20 were retained. The genotype of an individual at a given position was called if there was at least five-read coverage at the position, the frequency of the allele at the position was > 5%, and the allele was called at least twice. If more than one allele passed this filtering (heterozygous) the individual’s genotype at the position was called missing data. Across the entire sample set, SNP positions with ≥80% missing data at a AGPv4 position and ≥ 95% missing data at a Trinity transcript position were discarded.

An additional quality control was performed by manual inspection of pedigree information of the inbreds compared to their relative position on a phylogenetic tree created as described previously [38]. The phylogenetic position of one inbred line was inconsistent with the pedigree information. This inbred was removed from the dataset. Missing SNP calls were imputed using fastPHASE version 1.4.0 [108]. The -H flag was set to − 3 to impute without performing phasing, and default settings were used otherwise. Imputation accuracy was evaluated for 56 inbred lines by comparing imputed SNP calls to high quality SNPs from resequencing data. The imputation accuracy was defined as the proportion of imputed sites that matched the resequencing data.

### Calling variants from whole genome resequencing

Paired-end whole genome resequencing data was collected from a previous study [109] of 62 maize accessions, of which, 56 accessions are contained within the WiDi-942 panel. Reads were cleaned of barcode-specific adapter contamination and Illumina universal adapter contamination with Cutadapt version 1.13 [92]. Bases below a sliding window average quality score of 20 were then removed with Sickle [110]. Reads below 20 bp following adapter and quality trimming were discarded. When one read of a pair failed quality thresholds, its mate was retained for mapping as a single-end read. Cleaned reads were mapped to the B73 v4 reference assembly using Bowtie2 version 2.3.0 [91]. To account for the expected nucleotide diversity in maize, the seed length during mapping was set to 12 bp. Multiply mapping reads and duplicate reads were removed with SAMTools version 1.4 [107] and Picard Tools version 2.9.2 [111].

Variant calls were generated with the GATK HaplotypeCaller version 3.7.0 [112, 113]. A prior on nucleotide diversity of 0.02 was used. The resulting VCF file was filtered to remove length polymorphisms and multialleleic SNPs. A series of per-site and per-genotype filters were then applied. Variant sites with a QUAL score of less than 20 or greater than 5% observed heterozygosity were discarded. If an individual genotype was supported by less than 3 reads, greater than 50 reads (100 reads in the case of PH207, PHG35, PHG47, and PHJ40 to accommodate the higher average sequencing depth), or was a heterozygous call, it was set to missing. Sites that were monomorphic after heterozygosity and depth filtering were then removed.

### Field phenotypic data

Phenotypic data were collected for a subset of the WiDiv-942 panel planted in 2008, 2009, 2010, 2013, 2014, and 2015. Inbred lines were grown at the West Madison Agricultural Research Station in Madison, WI in one-row plots (3.8 m long and 0.76 m apart) with a density of 61,000 plants per hectare. Flowering time was recorded in 2013, 2014, and 2015 as growing days from planting until at least 50% of the plants within a plot shed pollen. Plant height, stalk diameter, and stalk anatomical measurements were collected from a randomized complete block design with two replications and three samples per plot. Plant height was measured from the soil surface to the node of the flag leaf in 2008, 2009, and 2010. Stalk diameter, rind thickness, vascular bundle area, and vascular bundle density were measured from scanned images of the third above ground internodes, which were harvested approximately 45 days after flowering, in 2013 and 2014. In addition to image-based data, stalk diameter of the third lowermost internode was measured manually in the field in 2008, 2010, and 2015. Cob size measurements of transgenic lines and their wild siblings were obtained from scanned images of primary cob, as described by [114]. Internode length was estimated by dividing plant height by node number. Tassel branch number was measured manually by counting the number of branches per tassel, and stover yield was measured from dry weight of stalk, leaf, tassel, and primary cob.

### Processing stem cross section images

Images of internode cross sections were obtained according to [30]. Custom software determined stalk diameter by finding the two points furthest apart on the outer boundary of the stalk. Calculation of vascular bundle area started with locating the centers of the vascular bundles as described in the aforementioned paper. A 2D-Gasussian distribution was fit to the brightness within a bundle. With the peak amplitude at, or close to the found center, the rims of the bundle were determined as the location at which the height of the distribution dropped beneath a predefined threshold. The two main axes, along which the distribution stretched, were determined using covariance matrix analysis. The radii of the two axes were used to calculate the area of the individual bundle as described previously [115]. Rind thickness and vascular bundle area measurements were obtained from the custom developed image processing software described by [30].

### Evaluation of phenotypic data

The heritability for each trait was estimated using the following formula: ${\hat{h}}^2=\frac{{\hat{\sigma}}_G^2}{{\hat{\sigma}}_G^2+\kern0.5em \left({\hat{\sigma}}_{GE}^2/y\right)+{\hat{\sigma}}_e^2/ ry}$, as described by [116], where ${\widehat{\sigma}}_G^2$ is genetic variance, ${\widehat{\sigma}}_{GE}^2$ is genotype-by-environment interaction variance, ${\widehat{\sigma}}_e^2$ is error variance, y is number of years, and *r* is number of replications. The analysis of variance (ANOVA) was performed by fitting a mixed linear model via restricted maximum likelihood method (REML), using the “lme4” package [117] in R [118]. The overall mean was included in the model as the fixed effect, whereas the effect of genotype, environment, replication, genotype by environment interaction, and replication nested within environment were considered random. The model assumptions (normality of residuals, homogenous variance of residuals, and normality of random effects) were assessed for each model. BLUP values for each trait were calculated using “ranef” function within the “lme4” package. Correlations analysis was performed using “PerformanceAnalytics” package [119].

### Genome-wide association analysis

GWAS were performed using BLUP values obtained from the mixed linear model implemented in the FarmCPU package [40] in R. SNPs with minor allele frequency less than 0.05 were discarded by setting “MAF.calculate” parameter to “TRUE”. Optimal bin size and quantitative trait nucleotide (QTN) number were selected for each trait based on the model fitness by inspecting Q-Q plots. The FarmCPU default fixed combination of bin size and QTN number were used in GWAS of plant height. For GWAS of the remaining traits, “method.bin” parameter was set to “optimum”. Candidate genes corresponding to GWAS hits were detected using physical position of genes based on AGPv4 reference sequence assembly [94]. Annotation of candidate genes was obtained from MaizeGDB [120], BLAST alignments to *A. thaliana*, matches to Pfam [121], and Phytozome v12.1 [103].

### Transformation

A pENTR SD/D-TOPO Gateway™ clone of *Zmm22* cDNA (GenBank ID KJ726934) was developed as described previously [122]. The cDNA clone was inserted into Gateway pDONR Zeo™ entry vector (Invitrogen, Catalog number: 12535035). The accuracy of the cloned sequence was confirmed by Sanger sequencing. The cDNA fragment was subsequently subcloned into the expression vector pANIC 6D (TAIR accession: Vector:6530479725) [123]. *Agrobacterium* strain AGL1 containing the expression vectors was used to transform immature embryos of maize hybrid Hi II kernels as described by [124].

### Evaluation of transgenic plants

Hi II hybrids transformed with *Zmm22* over-expression vectors were crossed to B73 inbred lines to produce T_1_ seeds. T_1_ transgenic plants were backcrossed to B73 to develop T_2_ seeds. T_1_ and T_2_ seeds were planted in 2015 and 2016, respectively, using one-row plots and two replications in a transgenic isolation field at the West Madison Agricultural Research Station. The first two plants from the beginning and end of each row were excluded from phenotypying to avoid the border effect. Seeds were screened for the red fluorescent protein using a LEICA M165 FC fluorescent microscope. To screen for bialaphos resistance, a marked area on the 5th or 6th leaf of plants was painted with 1% (*v*/v) Basta™ herbicide. Plants were scored for herbicide resistance 10 days after Basta application. For sq-PCR analysis, total RNA was isolated from the 9th leaf using Invitrogen PureLink® RNA Mini Kit (Catalog number: 12183018A) and purified by treatment with Invitrogen DNase I amplification grade. First strand cDNA was synthesized from Poly (A)^+^ RNA using Invitrogen SuperScript® III First-Strand Synthesis System (Catalog number: 18080051). A 150 bp segment of *Zmm22* cDNA was amplified with 5’-ACTCCTCCTCCAGCATAGAAG-3′ and 5’-TGTTTAGACCATGTTGCGATCTCC-3′ primers. Eukaryotic initiation factor 4A (*EIF4A)* gene was selected as the reference gene [125]. PCR was performed using Promega PCR master mix (Catalog number: M7501). The PCR condition included an initial 5 min of denaturing at 94 °C, 25 and 35 cycles of 94 °C for 10 s, 59 °C for 30 s, and 72 °C for 30s, followed by 5 min final extension at 72 °C. PCR products were visualized on 1.5% agarose gel electrophoresis.

## Additional files

Additional file 1: Pedigree information, subpopulations, stalk traits BLUP values, and information of RNA-Seq reads for the WiDiv-942 population. (XLSX 294 kb)

Additional file 2: Q-Q plots assessing the fitness of K model for GWAS of stalk traits. (PPTX 1508 kb)

Additional file 3: Manhattan plot of GWAS result for vascular bundle area. (PPTX 294 kb)

Additional file 4: LD plots of genomic regions containing significant SNPs and adjacent candidate genes. Significant SNPs are shown in red boxes. Each LD block contains all of the SNPs within a specific gene. (PPTX 4069 kb)

Additional file 5: Expression profile of detected candidate genes based on the maize gene atlas dataset [42, 43] (XLSX 3497 kb)

Additional file 6: Gel electrophoresis showing sqPCR results of a sample of transgenic lines (T) and non-transgenic siblings (C). (PPTX 226 kb)

Additional file 7: Comparison of the average of plant height and stalk diameter for two *Zmm22* over-expression events and their non-transgenic siblings in T_1_ generation. Traits were measured from one-row plots in replicated field trials. “*” indicates significance at *p* < 0.05. (XLSX 9 kb)

Additional file 8: Correlation between flowering time and stalk traits. Spearman correlation was calculated between BLUP values. (PDF 354 kb)

## Abbreviations

ABC: ATP-binding cassette
AGPv4: Version 4 maize B73 reference sequence assembly
BLUP: Best linear unbiased prediction
BSSSC0: cycle 0 of Iowa stiff stalk
Cdk: Cyclin-dependent kinase
DUF: Domain with unknown function
ExPVP: Expired plant variety protection
GEM: Germplasm enhancement of maize
GWAS: Genome-wide association studies
IDT: Iodent
K: Kinship
LD: Linkage disequilibrium
Lhcb: Light harvesting chlorophyll binding
MDR/PGP: Multi drug resistance class of P-Glycoproteins
NSS: Non-stiff stalk
PE: Pectinesterase
Q-Q: Quantile-quantile
QTL: Quantitative trait loci
QTN: Quantitative trait nucleotide
RFP: Red fluorescent protein
SNP: Single nucleotide polymorphism
sqPCR: Semi-quantitative PCR
SS: Stiff stalk
TLK: Tousled-like kinases
WiDiv: Wisconsin Diversity
