# Supplementary material for: Genome-wide association analysis of stalk biomass and anatomical traits in maize
Source: BMC Plant Biol. 2019 Jan 31;19:45. doi: 10.1186/s12870-019-1653-x (PMC6357476; doi:10.1186/s12870-019-1653-x)
Supplement: Supplementary file 2 — Q-Q plots assessing the fitness of K model for GWAS of stalk traits. (PPTX 1508 kb) [file 12870_2019_1653_MOESM2_ESM.pptx]

## Slide 1
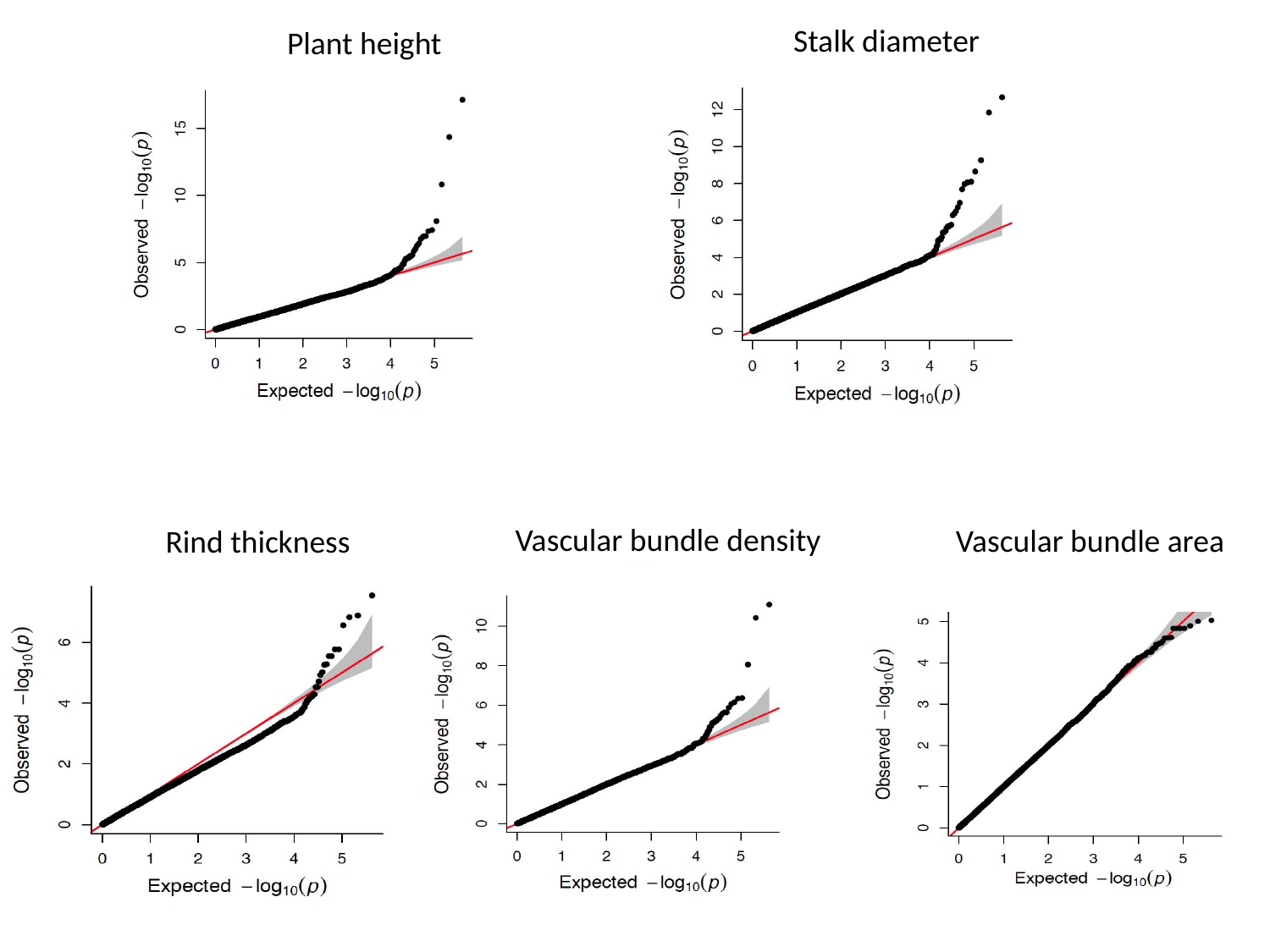

Stalk diameter
Plant height
Vascular bundle density
Vascular bundle area
Rind thickness
