# Supplementary figures and images for: Genome-wide association analysis of stalk biomass and anatomical traits in maize
Source: BMC Plant Biol. 2019 Jan 31;19:45. doi: 10.1186/s12870-019-1653-x (PMC6357476; doi:10.1186/s12870-019-1653-x)

## Slide 1
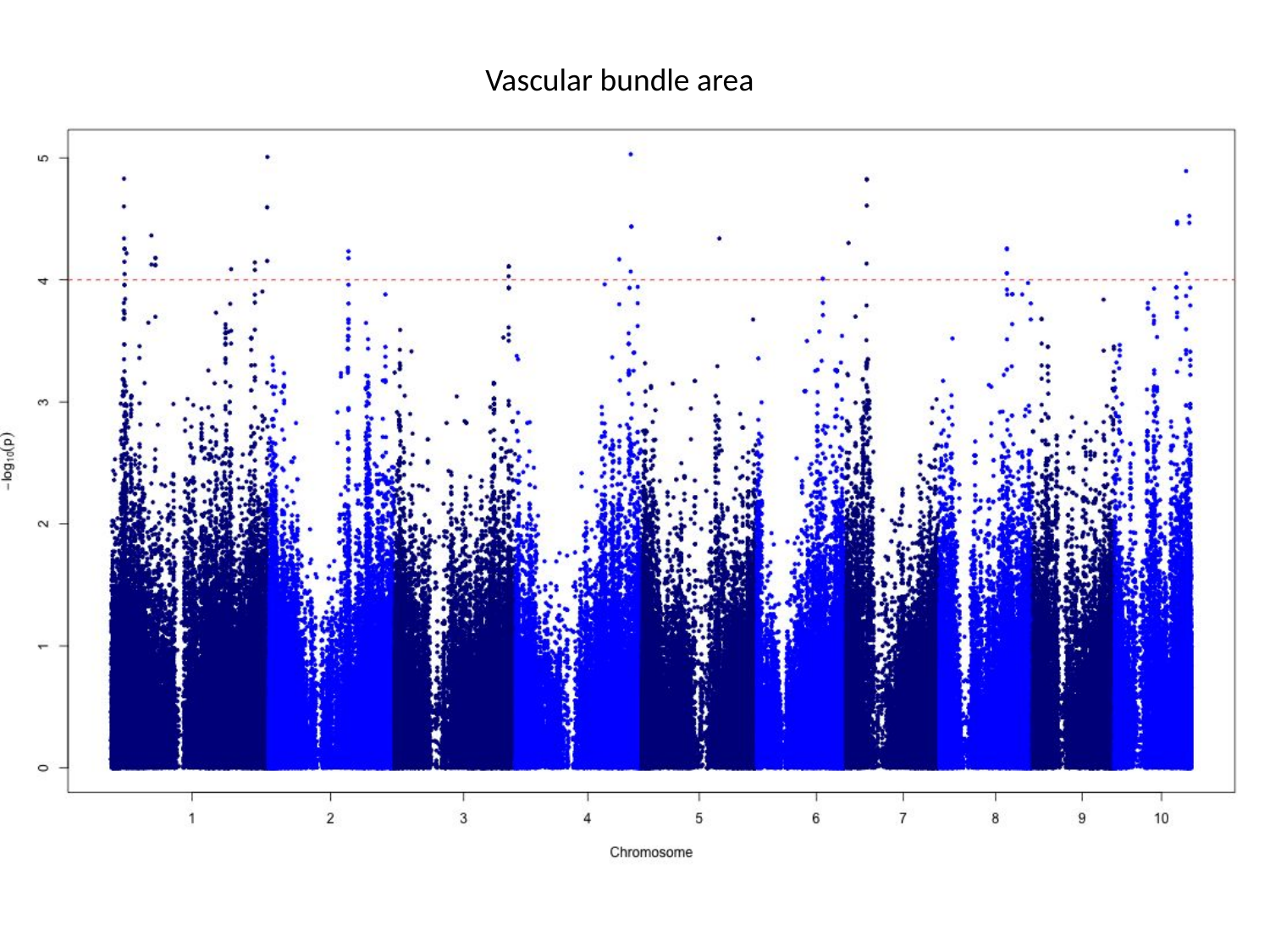

Vascular bundle area

Supplement: Supplementary file 3 — Manhattan plot of GWAS result for vascular bundle area. (PPTX 294 kb) [file 12870_2019_1653_MOESM3_ESM.pptx]

## Slide 1
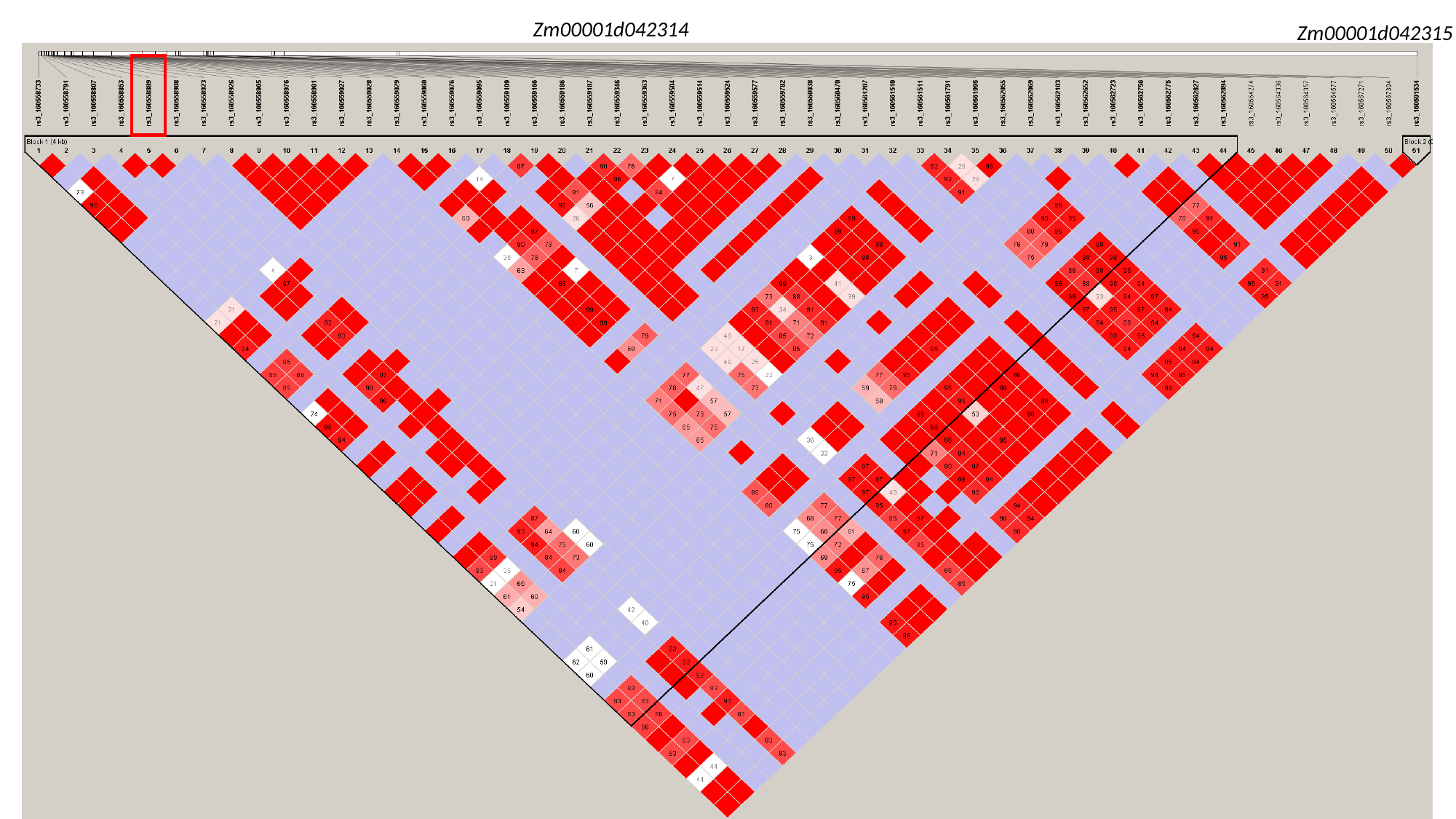

Zm00001d042314
Zm00001d042315

## Slide 2
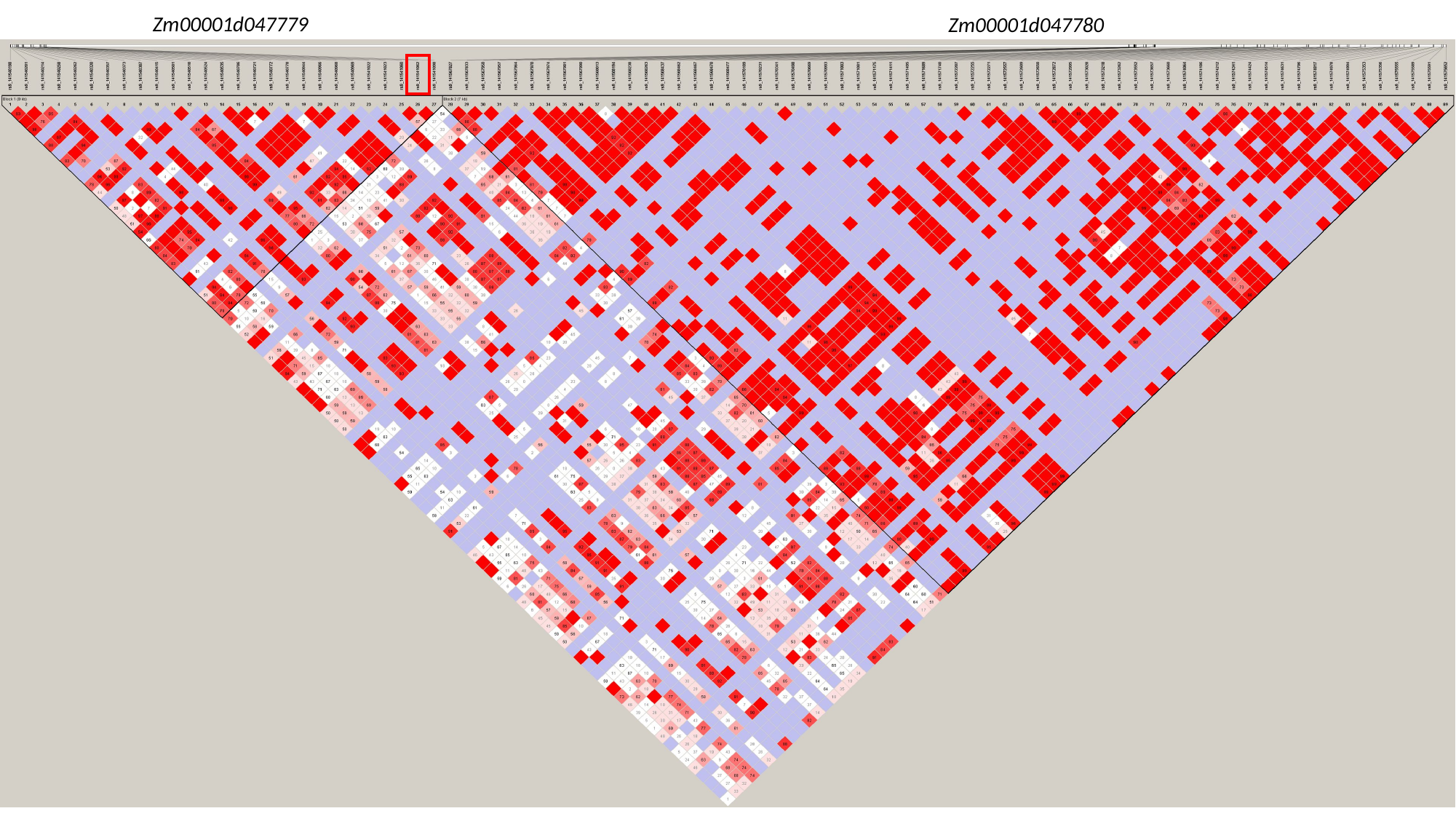

Zm00001d047779
Zm00001d047780

## Slide 3
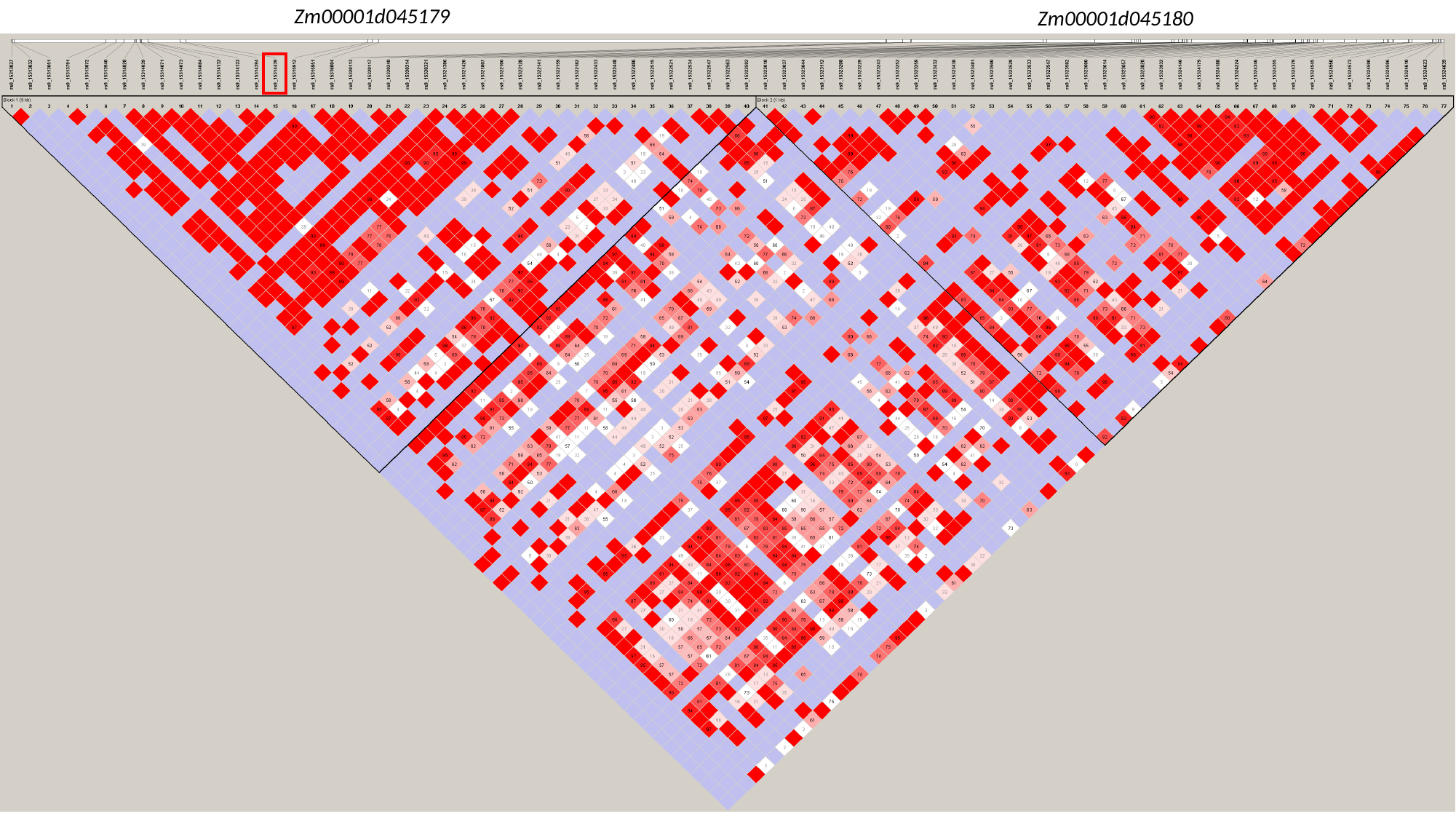

Zm00001d045179
Zm00001d045180

## Slide 4
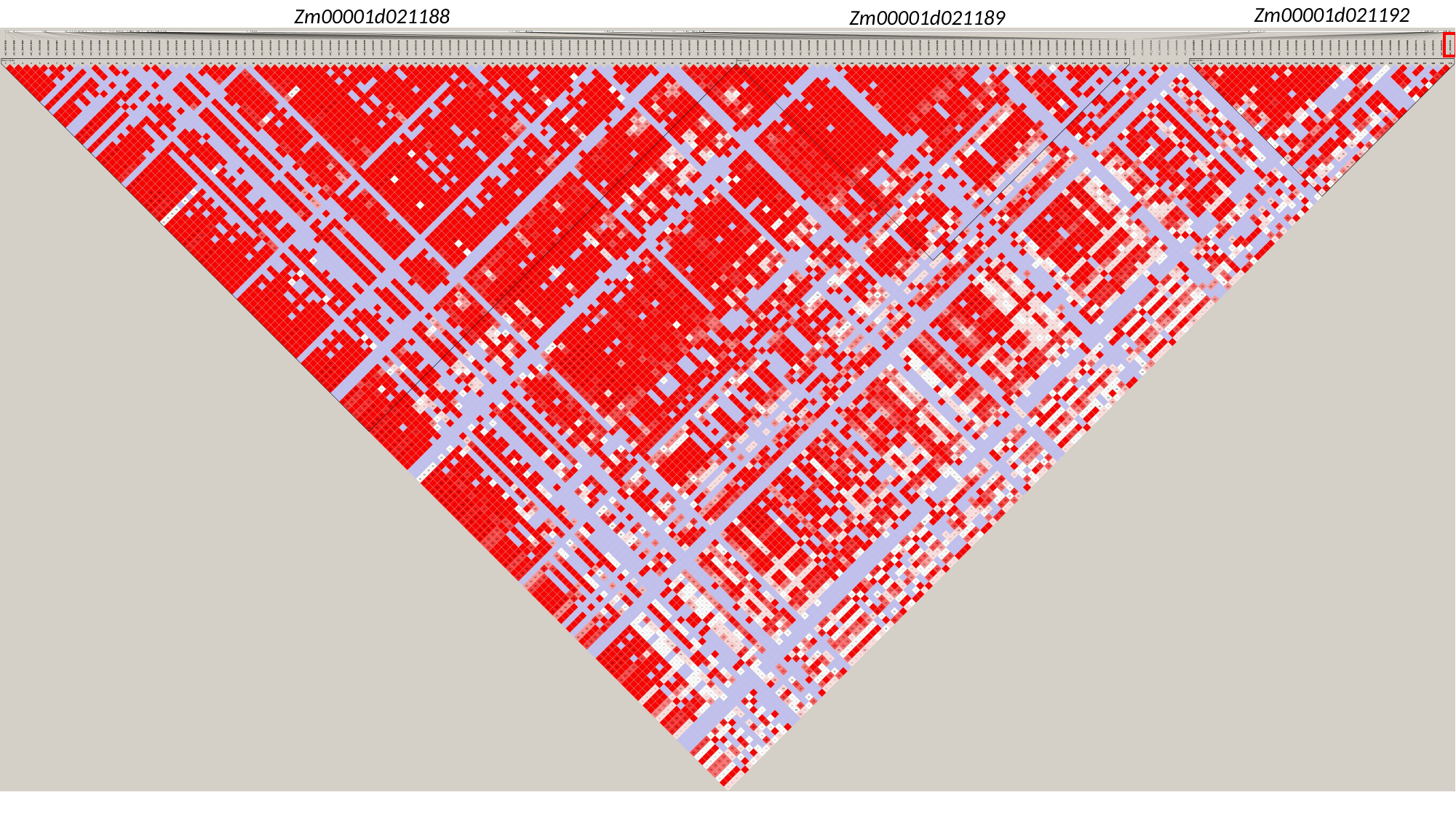

Zm00001d021192
Zm00001d021188
Zm00001d021189

## Slide 5
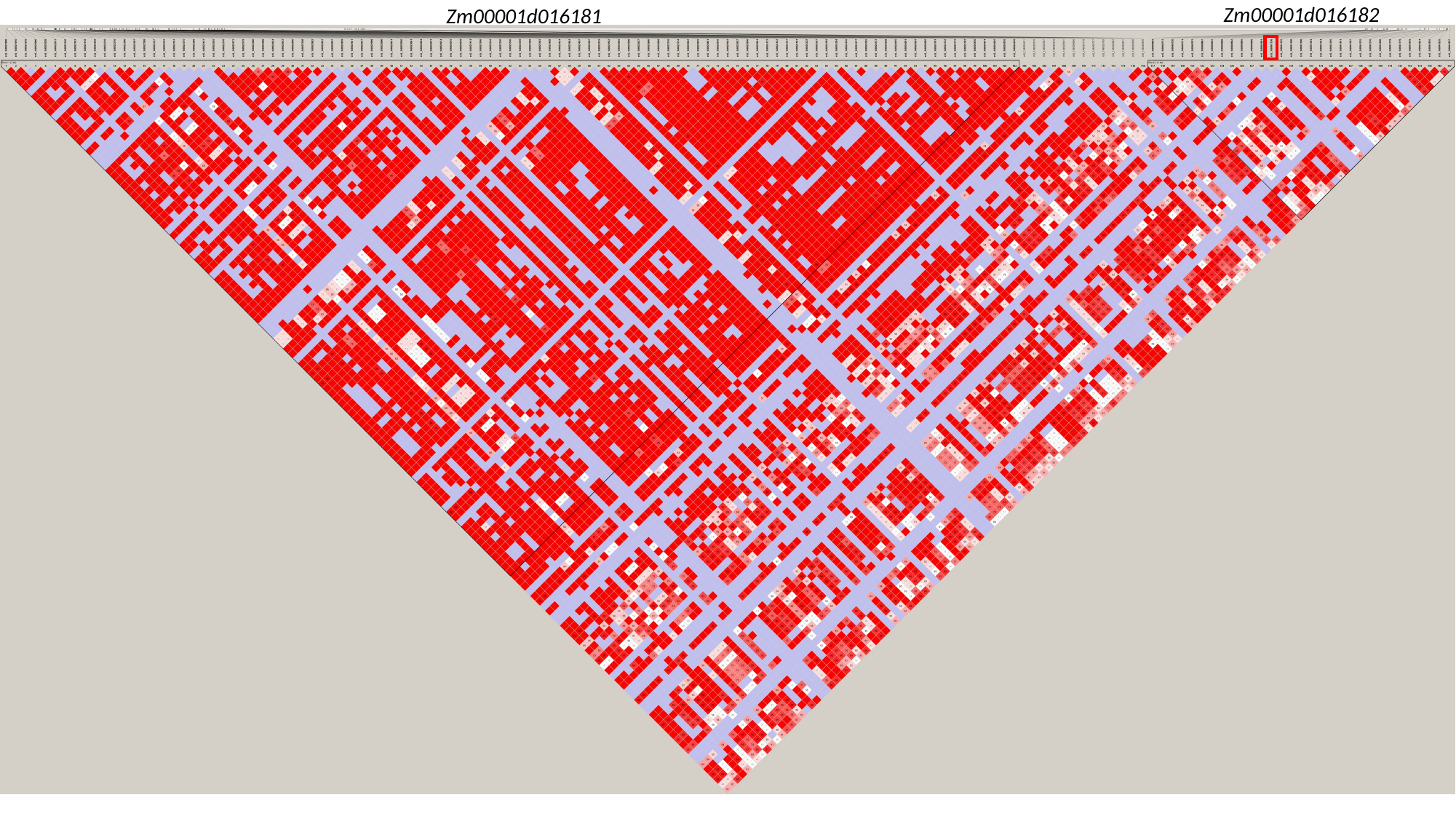

Zm00001d016182
Zm00001d016181

Supplement: Supplementary file 4 — LD plots of genomic regions containing significant SNPs and adjacent candidate genes. Significant SNPs are shown in red boxes. Each LD block contains all of the SNPs within a specific gene. (PPTX 4069 kb) [file 12870_2019_1653_MOESM4_ESM.pptx]
