# Supplementary material for: Genome-wide association analysis of stalk biomass and anatomical traits in maize
Source: BMC Plant Biol. 2019 Jan 31;19:45. doi: 10.1186/s12870-019-1653-x (PMC6357476; doi:10.1186/s12870-019-1653-x)
Supplement: Supplementary file 6 — Gel electrophoresis showing sqPCR results of a sample of transgenic lines (T) and non-transgenic siblings (C). (PPTX 226 kb) [file 12870_2019_1653_MOESM6_ESM.pptx]

## Slide 1
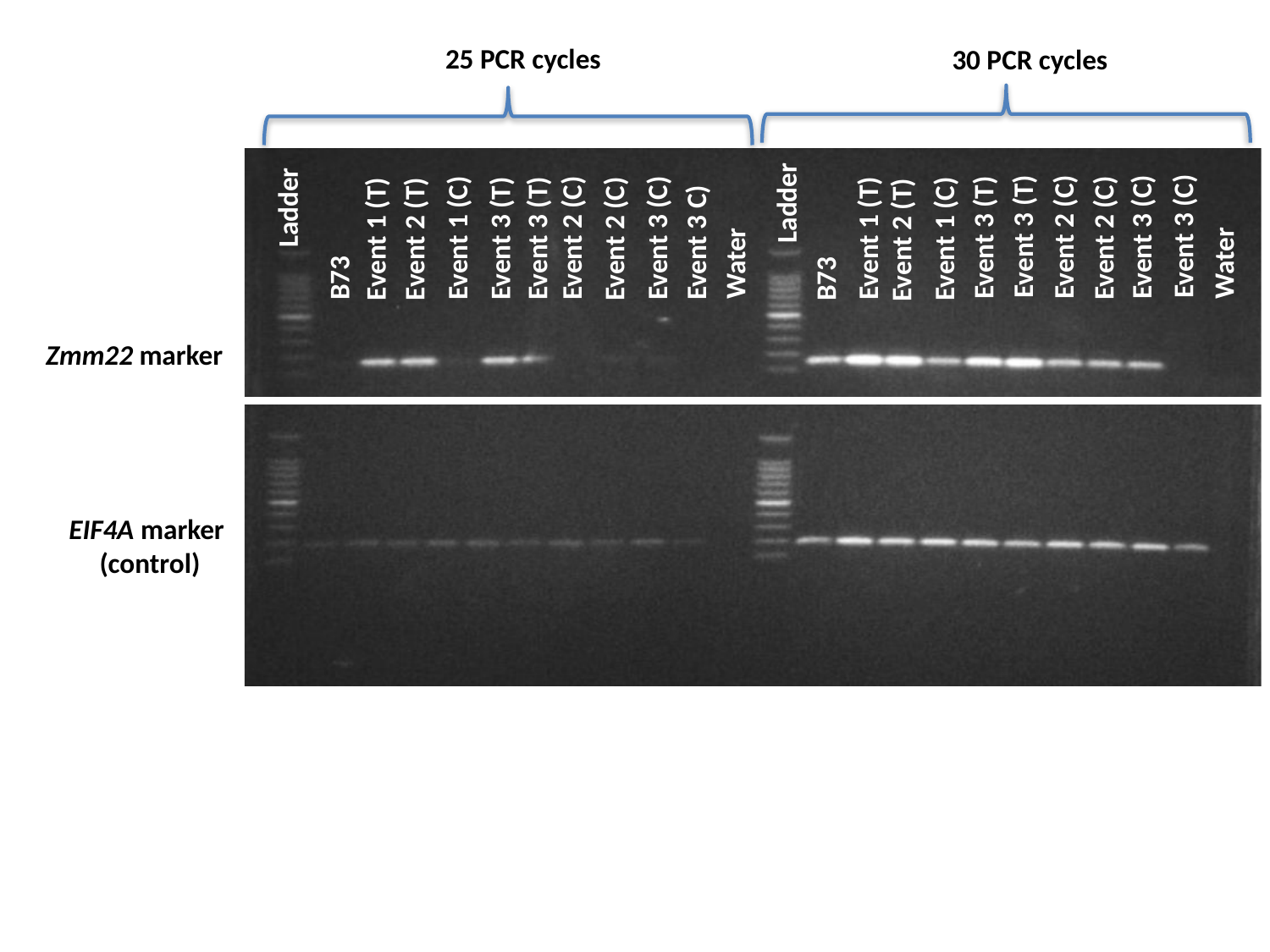

25 PCR cycles
30 PCR cycles
Event 1 (T)
Water
B73
Ladder
Ladder
Zmm22 marker
EIF4A marker
 (control)
Event 1 (C)
Event 1 (C)
Event 3 (C)
Event 3 (T)
Event 3 (C)
Event 2 (C)
Event 3 (T)
Event 3 (C)
Event 2 (C)
Event 2 (C)
Event 1 (T)
Event 3 (T)
Event 3 (T)
Event 2 (C)
Event 2 (T)
Event 2 (T)
Event 3 C)
Water
B73
