# Supplementary material for: Genome-wide association analysis of stalk biomass and anatomical traits in maize
Source: BMC Plant Biol. 2019 Jan 31;19:45. doi: 10.1186/s12870-019-1653-x (PMC6357476; doi:10.1186/s12870-019-1653-x)
Supplement: Supplementary file 8 — Correlation between flowering time and stalk traits. Spearman correlation was calculated between BLUP values. (PDF 354 kb) [file 12870_2019_1653_MOESM8_ESM.pdf]

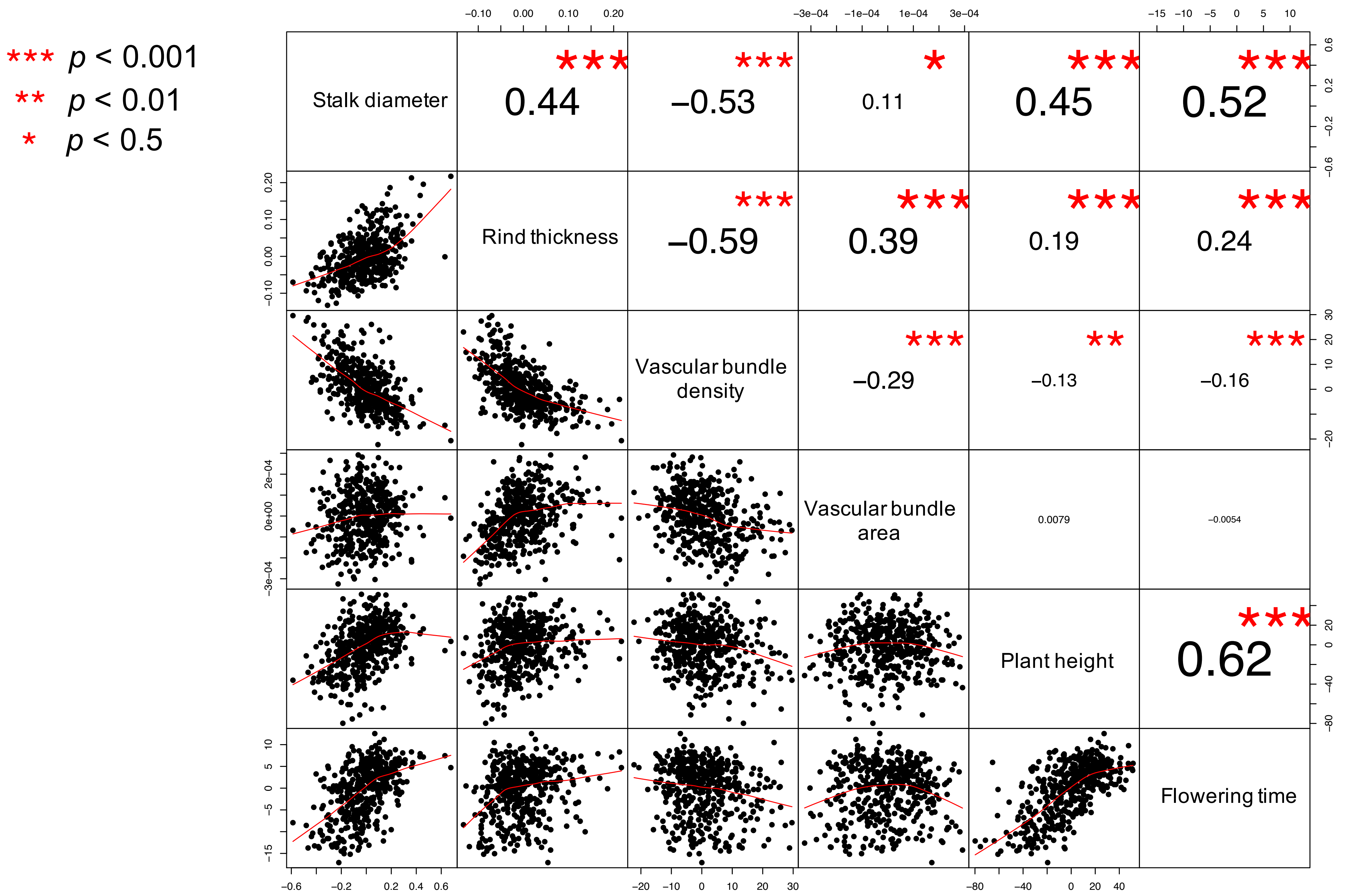

*** p < 0.001
** p < 0.01
* p < 0.5
Stalk diameter
Rind thickness
Vascular bundle density
Vascular bundle area
Plant height
Flowering time
0.44
−0.53
0.11
0.45
0.52
−0.59
0.39
0.19
0.24
−0.29
−0.13
−0.16
0.0079
−0.0054
0.62
